# 1223. The Impact of the COVID-19 Pandemic on the Prevalence of Multidrug-resistant Organisms in Veterans Affairs Nursing Homes

**DOI:** 10.1093/ofid/ofac492.1055

**Published:** 2022-12-15

**Authors:** Monirul I Sajib, Florence M Ford, George Psevdos

**Affiliations:** Stony Brook University Hospital, West Babylon, New York; Northport VA, Northport, New York; Northport VA Medical Center, Northport, New York

## Abstract

**Background:**

Multidrug-resistant organisms (MDRO) in long-term care facilities are prevalent and pose a major health concern for their residents. During the COVID-19 pandemic, infection control measures were justifiably heightened in every aspect of health care, including nursing homes. There are reports depicting decreasing prevalence of MDRO in hospital settings during the pandemic. We compared the prevalence of MDRO in our facility’s nursing homes in the two-year period before vs. the two years of the pandemic.

**Methods:**

Northport Veterans Affairs Medical Center provides long-term nursing home care structured as community living centers including mental health and hospice care; a 139 total bed capacity. A retrospective review of culture data collected by infection control preventionists comparing the prevalence of MDROs between 1 March 2018 to 28 February 2022 was performed. Data included: Nasopharyngeal MRSA swabs, urine, wound, blood, sputum cultures, *C. difficile* toxin and PCR assays. MDRO included ESBL *E. coli*, *K. pneumoniae*, *P. mirabilis*, MDR *Pseudomonas spp* (resistance to 2/3 antibiotics: cefepime/piperacillin-tazobactam/ciprofloxacin), carbapenem-resistant *Pseudomonas spp*, and vancomycin-resistant *Enterococcus spp*.

**Results:**

There were 75209 bed days of care from 1 March 2018 to 28 February 2020 vs. 77531 from 1 March 2020 to 28 February 2022. The MRSA rate per 1000 patient days decreased from 4.98 pre-COVID to 2.70 during-COVID, P < 0.001. Similarly, there was a decrease in *C. difficile* 0.69 vs. 0.13, P< 0.001, ESBL *E. coli* 0.53 vs. 0.51, P< 0.001 and *S. maltophilia* (no cases during COVID). There was an increase in ESBL *K. pneumoniae* 0.51 vs. 0.63, P< 0.001, MDR *Pseudomonas* 0.05 vs. 0.49, P< 0.001, carbapenem-resistant *Pseudomonas* 0.026 vs. 0.077, P< 0.001 and VRE 0.22 vs. 0.31, P< 0.001. There were no *Candida auris* or *Acinetobacter spp* detected in the study period.

MDRO occurrences before and during COVID-19 pandemic

**Figure:**
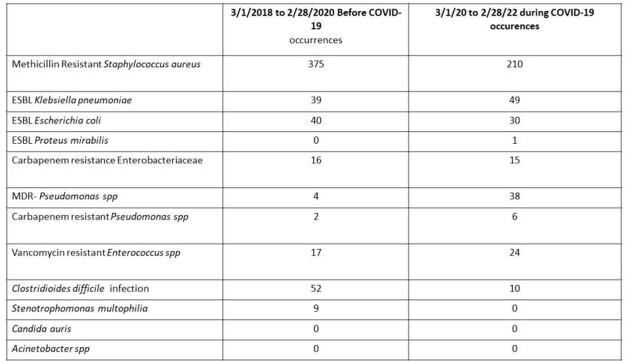

**Conclusion:**

While increased awareness and implementation of infection control measures during the years of the COVID-19 pandemic led to decrease in certain infections in our nursing homes, like *C. difficile*, a surprising uptick in ESBL *K. pneumoniae* and MDR *Pseudomonas* was noted. This is a concerning trend that merits further study to identify molecular factors and increase stewardship efforts in diligent use of carbapenems.

**Disclosures:**

**All Authors**: No reported disclosures.

